# Radiofrequency Ablation of Adenoma Sebaceum

**DOI:** 10.4103/0974-2077.44166

**Published:** 2008

**Authors:** Swaroop MR, Nischal KC, Rajesh Gowda CM, Umashankar NU, Basavaraj HB, Sathyanarayana BD

**Affiliations:** *Department of Dermatology, Adichunchanagiri Institute of Medical Sciences and Research, BG Nagar, Karnataka, India*; 1 *Department of Radiology, Adichunchanagiri Institute of Medical Sciences and Research, BG Nagar, Karnataka, India*

**Keywords:** Adenoma sebaceum, radiofrequency, tuberous sclerosis, disfigurement

## Abstract

Adenoma sebaceum is one of the diagnostic features of tuberous sclerosis. Histologically, they are angiofibromas that occur over the central part of the face and hence, cause a major cosmetic disfigurement. Different forms of ablative treatments including laser ablation have been used for the treatment of this condition. Laser treatment is expensive and any form of treatment for adenoma sebaceum is not a one-time procedure but is a recurring process as the condition is genetic in aetiology. It is therefore appropriate to use a cheap and easily available modality, particularly in the Indian scenario. We hereby report a case of tuberous sclerosis in whom we ablated the lesions by radiofrequency technique with acceptable results.

## INTRODUCTION

Adenoma sebaceum, pathognomonic of tuberous sclerosis, are tiny angiofibromas which commonly occur over central part of face. This causes severe disfigurement and lowers the morale of patients. Various modalities of treatment, *viz*., lasers, cryosurgery, dermabrasion have been used with varying results.[1–8] Recurrence after treatment is common and hence a need for inexpensive, but safe and efficient treatment. There are several reports of laser ablation of these growths,[7,8] but using laser for all patients is not feasible in India due to the expenditure incurred, and the expertise and infrastructure required. Radiofrequency ablation is a safe, economical procedure and has been known to cause less scarring than other ablative methods such as electrocautery. It has been used for treating various neoplasms with effective results.[9–11]

## CASE REPORT

A 20 year-old male presented with asymptomatic, small, raised lesions on the face for past five years. Lesions gradually increased in size as well as number over the years, causing embarrassment and low self-esteem. Enquiry produced a history of scholastic backwardness but none suggestive of epilepsy or similar affliction of other family members.

**Figure 1:**
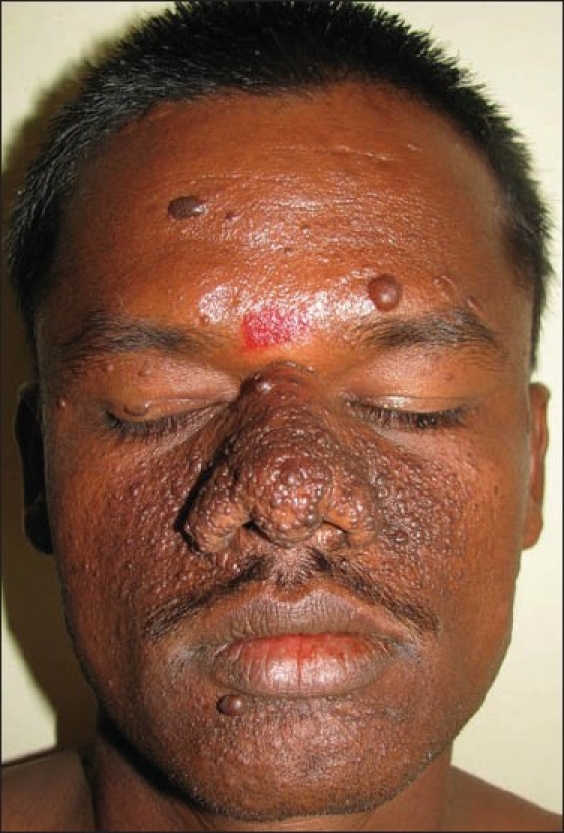
Skin-coloured to hyperpigmented papules and nodules on the nose, cheeks along with a hyperpigmented peau d’ orange plaque on the right forehead

Examination revealed that the patient had multiple, tiny, hyperpigmented to skin-coloured papules and nodules on forehead, upper eyelids, dorsum of nose, cheek, and chin [Figure 1]. He also had Koenon’s tumor, shagreen patch, and dental pits; there was no history of hypertrophic scars or keloids. Presence of periventricular tubers in a CT scan of the brain confirmed the diagnosis of tuberous sclerosis.

**Figure 2:**
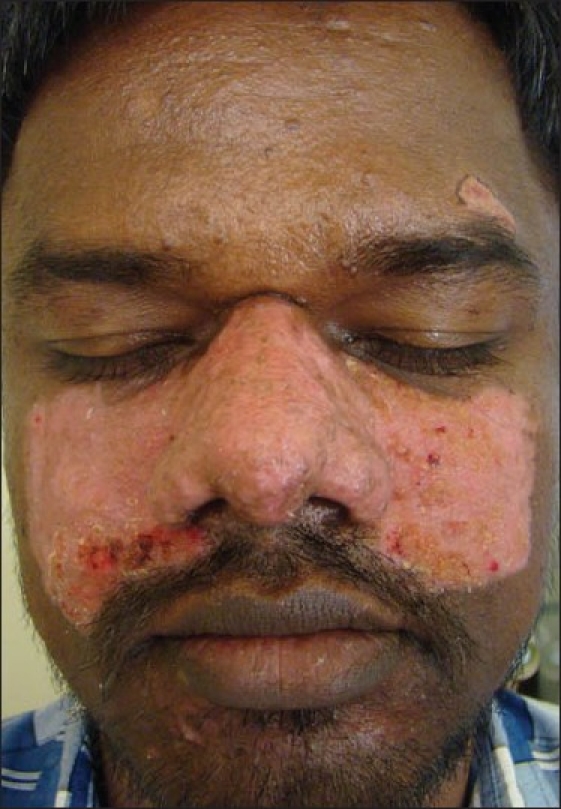
Epithelialised wound with areas of crusting one week after the procedure

**Figure 3 A and B:**
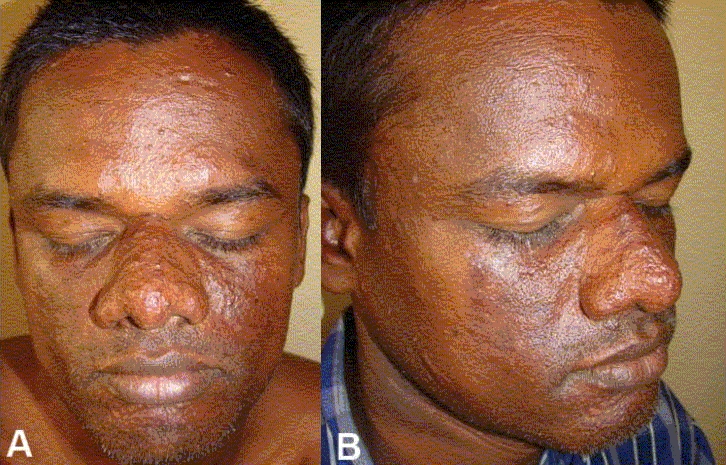
Prominent butterfly postinflammatory hyperpigmentation along with dilated follicular ostia

As the patient could not afford laser treatment for the adenoma sebaceum, the cheaper option of radiofrequency ablation was considered. The radiofrequency procedure was done under local anaesthesia by using Megasurg Gold™ (M/s Dermaindia) with a frequency 0.2– 2.93 MHz, 230 volts, using both cut (70% cut, 30% coagulation) and coagulation (60% coagulation, 40% cut) modes.[12] Local anaesthesia over the dorsum of the nose and cheeks was achieved by using a bilateral infraorbital nerve block using 2% lignocaine. Lesions on the forehead and chin were few and were anaesthetized by local infiltration of 2% lignocaine. Ablation was done initially under the cutting mode to flatten the skin and later, under the coagulation mode to smoothen the skin further and control the bleeding. After the procedure, dressing was done with antibiotic-paraffin tulle which was changed every day. Systemic antibiotics and analgesics were administered for a week. The mild crusting [Figure 2] which was collected initially was removed with fine-tipped Adson’s forceps and hydrogen peroxide. Ablated wound healed with mild hyperpigmentation in a butterfly pattern [Figures 3A and B] which was acceptable to the patient. Slight prominence of follicular ostia was evident on the dorsum of the nose; there was no evidence of scarring.

## DISCUSSION

The term ‘adenoma sebaceum’ is a misnomer as it is not related to the sebaceous glands, but is a benign tumour with both angiomatous and fibrous components. Adenoma sebaceum starts appearing by 5–10 years of age, and gradually increases till puberty, after which it is commonly found to stabilize. The papules commonly involve the central face, bilaterally and symmetrically covering the glabellar area, the dorsum of nose, adjoining cheeks, upper lip, and chin. However, there are reports of unilateral or segmental distribution of adenoma sebaceum.[13] Angiofibromas similar to adenoma sebaceum have been reported in multiple endocrine neoplasia I.[14] Although there are no reports of malignant transformation, they are of concern due to the cosmetic disfigurement. Hence, patients frequently seek treatment and it is necessary to treat these growths.

Various techniques for ablation of tumors include shave excision with cultured epithelial autografts[1] or dermabrasion,[2–4] cryotherapy,[5] lasers [argon laser,[6] carbondioxide laser,[7] and carbondioxide resurfacing with fibrin sealing.[8] There are various reports of ablation of adenoma sebaceum by lasers with varied results. Carbon dioxide lasers are commonly employed for ablation. Lasers are an expensive treatment for the patients and incur a huge investment in the form of infrastructure and instrument for the treating dermatologists.

The radiofrequency machine converts wall outlet alternating current to low amperage, high voltage current which generates high frequency (500–4000 KHz) oscillating radiowaves termed as Sine waves. Pure sine waves (cut mode) are employed for cutting and dampened waves (coagulation mode) for ablative procedures where there is a risk of bleeding.[15] Energy generated due to the passage of radiofrequency waves through the tissue causes destruction of tissues. Lateral spread of energy is minimal with a radiofrequency machine and hence, it gives better cosmetic results than electrocautery.

In dark-skinned patients, dermatosugical procedures are always fraught with the risk of postinflammatory hyperpigmentation.[16] In our case, the patient developed hyperpigmentation over both the cheeks and nose. As the patient was a laborer, nonadherence to photoprotection could have contributed for hyperpigmentation. In addition to this, there was slight prominence of the follicular opening, which is more commonly seen due to dermabrasion. Another disadvantage with the treatment of adenoma sebaceum is the recurrence of these hamartomas. Belmar *et al*. have found that patients older than 20 years of age had less recurrence than younger patients.[17] There are no reports of ablation of adenoma sebaceum by radiofrequency in current literature. Radiofrequency is a safe, effective, and economical therapeutic option for treating adenoma sebaceum. However, recurrences may occur, and further repeated treatments may be necessary.
